# The Role of Cysteine Residues in Catalysis of Phosphoenolpyruvate Carboxykinase from *Mycobacterium tuberculosis*

**DOI:** 10.1371/journal.pone.0170373

**Published:** 2017-01-30

**Authors:** Iva Machová, Martin Hubálek, Martin Lepšík, Lucie Bednárová, Markéta Pazderková, Vladimír Kopecký, Jan Snášel, Jiří Dostál, Iva Pichová

**Affiliations:** 1 Institute of Organic Chemistry and Biochemistry of the Czech Academy of Sciences, Prague, Czech Republic; 2 Institute of Physics, Faculty of Mathematics and Physics, Charles University in Prague, Prague, Czech Republic; University of Delhi—South Campus, INDIA

## Abstract

*Mycobacterium tuberculosis* (MTb), the causative agent of tuberculosis, can persist in macrophages for decades, maintaining its basic metabolic activities. Phosphoenolpyruvate carboxykinase (Pck; EC 4.1.1.32) is a key player in central carbon metabolism regulation. In replicating MTb, Pck is associated with gluconeogenesis, but in non-replicating MTb, it also catalyzes the reverse anaplerotic reaction. Here, we explored the role of selected cysteine residues in function of MTb Pck under different redox conditions. Using mass spectrometry analysis we confirmed formation of S–S bridge between cysteines C391 and C397 localized in the C-terminal subdomain. Molecular dynamics simulations of C391-C397 bridged model indicated local conformation changes needed for formation of the disulfide. Further, we used circular dichroism and Raman spectroscopy to analyze the influence of C391 and C397 mutations on Pck secondary and tertiary structures, and on enzyme activity and specificity. We demonstrate the regulatory role of C391 and C397 that form the S–S bridge and in the reduced form stabilize Pck tertiary structure and conformation for gluconeogenic and anaplerotic reactions.

## Introduction

Tuberculosis (TB), one of the oldest human diseases, remains one of the biggest infectious killers worldwide despite the availability of drugs and vaccine. Latent *Mycobacterium tuberculosis* (MTb) infection is a major obstacle in global TB control and hinders complete eradication of the disease. Latency develops due to the ability of MTb to escape host defense mechanisms, such as reactive oxygen species and reactive nitrogen species. The metabolism of MTb is quite flexible, and the bacterium is capable of adapting to extremely different conditions and environments. The process of adaptation is a complex system of transition from an energy-consuming replicative state to a state of dormancy, characterized by suppression of metabolism to the minimum. The major transition trigger is likely a decrease of oxygen inside lung granulomas, where MTb can survive for decades. Hypoxia arrest inside macrophages induces increase in the level of NADH and NADPH cofactors in the MTb intracellular milieu and decrease in ATP level [1,2]. Transcriptional and metabolomics studies have indicated induction of the DosR/S [3] system and KstR repressor [4] and upregulation of several enzymes involved in central carbon metabolism, including isocitrate lyase, phosphofructokinase B, phosphoenolpyruvate carboxykinase (Pck), and others [1,5,6] during bacterial slow growth. Upregulation of the Pck gene has been detected in non-growing bacilli during mouse infection [7]. This rearrangement of metabolic routes leads to the synthesis of oxaloacetate (OAA) by anaplerotic fixation of CO_2_. Pck is a GTP-dependent enzyme that catalyzes the reversible step of phosphoenolpyruvate (PEP) production from oxaloacetate (OAA). Pck activity is preferentially associated with the gluconeogenic production of PEP, but under hypoxia-triggered non-replicating conditions, it also effectively catalyzes the anaplerotic reaction, resulting in OAA production in MTb [1,8]. In our previous study, we found that the specificity of MTb Pck is strictly regulated by reducing conditions in the anaplerotic reaction [9]. While synthesis of PEP by MTb Pck does not require reducing conditions, OAA is synthesized only in the presence of a reducing agent in the anaplerotic reaction.

The MTb Pck sequence contains nine cysteine residues, six of them (C49, C89, C119, C131, C198, and C230) are situated in the N-terminal domain (C89, C119, and C198 are located in random coil loops; C49 and C230 in α-helices, C131 in a β-sheet), C391 and C397 are present in the C-terminal subdomain between the N- and C-terminal domains. The reactive cysteine C273 that is typical for the GTP-dependent Pck family is localized in the binding site within the C-terminal domain, close to the binding site for GDP/GTP [10]. Mutational analysis, however, showed that C273 residue is not essential for Pck function in either directions [9].

In this study, we investigated the role of cysteine residues that could potentially contribute to regulation of MTb Pck specificity by the change of reducing conditions. We identified critical cysteine residues in positions 391 and 397 that significantly contribute to protein stability and consequently to MTb Pck activity.

## Materials and Methods

### Cloning, expression, and purification of MTb Pck

Cloning and expression of MTb Pck has been previously described [9]. Pck (Rv0211) with an N-terminal His-tag in pET15b and all cysteine mutants were expressed in *E*. *coli* BL21(DE3). The purification process was performed in batches, followed by size exclusion chromatography. Harvested cells were lysed by multiple freeze-thaw cycles and addition of lysozyme. The cell supernatant was loaded onto Talon^®^ chromatography resin (Clontech) and gently agitated for 1 h on a shaker at 4°C to allow the His-tagged protein to bind the resin. After incubation, the resin was centrifuged at 700 × g for 5 min, and the supernatant was removed. The resin pellet was washed with 10-bed volumes of buffer (20 mM Tris-HCl, 300 mM NaCl, pH 8) and then with 10-bed volumes of buffer containing 10 mM imidazole. The Pck variants were eluted by sequentially increasing the imidazole concentration (100, 200, and 500 mM) in the elution buffer. Pck-containing fractions were filtered to remove traces of resin, concentrated, and purified on a HiLoad 16/60 FPLC (Superdex 75 pg, GE Healthcare) equilibrated with 20 mM Tris-HCl, pH 7.4, or 20 mM Tris-HCl, 150 mM NaCl, pH 7.4.

### Site-directed mutagenesis of C391 and C397

Site-directed mutagenesis was performed according to the QuikChange protocol by Stratagene^TM^ with minor modifications. The complementary primers (1 μg/ml) (see Table 1) were mixed in *Pfu* polymerase reaction buffer with 0.2 mM dNTPs and 50 ng plasmid template containing the Pck coding sequence. After addition of *Pfu* polymerase (2 U/reaction), the following cycling reaction (linear amplification reaction) was started: denaturation at 94°C (50 sec), annealing at 55°C (50 sec), and extension at 72°C (8 min), with 20 repeats. After the cycling reaction, *Dpn*I restriction enzyme was added, and the final mixture was subsequently incubated at 37°C for 12 h. The final product was transformed into DH5α ultracompetent cells, and individual colonies were used for sequencing.

**Table 1:** Primer sequences used for site-directed mutagenesis of MTb Pck.

Mutant	Forward primer: 5′-3′	Reverse primer: 5′-3′
C391A	CCGAACTCCCGGTACGCCACACCGATGTCGCAG	CTGCGACATCGGTGTGGCGTACCGGGAGTTCGG
C397A	ACACCGATGTCGCAGGCCCCGATCCTGGCCCCC	GGGGGCCAGGATCGGGGCCTGCGACATCGGTGT

### Enzyme activity measurement

Pck activity was determined as previously described [9]. To monitor the rate of the anaplerotic reaction (OAA formation), the Pck-catalyzed reaction was coupled with the subsequent reaction catalyzed by malate dehydrogenase from *Thermus flavus* (Sigma-Aldrich). The standard reaction mixture contained 100 mM HEPES-NaOH, pH 7.2, 100 mM KHCO_3_, 37 mM DTT, 2 mM PEP, 1 mM GDP, 2 mM MgCl_2_, 0.1 mM MnCl_2_, 2 U/ml MDH, and 0.25 mM NADH. Each reaction was started by addition of the essential divalent cations (Mg^2+^ and Mn^2+^). To monitor the gluconeogenic reaction (PEP formation), the Pck-catalyzed reaction was coupled with reactions catalyzed by pyruvate kinase (PK, Roche) and lactate dehydrogenase (LDH, Roche). The typical reaction mixture was composed of 100 mM HEPES-NaOH, pH 7.2, 0.3 mM OAA, 0.2 mM GTP, 2 mM MgCl_2_, 0.2 mM MnCl_2_, 10 mM DTT, 10 U/ml LDH, 3 U/ml PK, and 0.2 mM NADH. The reaction progress for both reactions was followed by monitoring the decrease in absorbance at 340 nm due to NADH oxidation to NAD^+^. The kinetic data were fitted using a nonlinear least squares regression analysis (SigmaPlot 11.0).

### Mass spectrometry analysis

To identify native disulfide-containing peptides, wt MTb Pck was digested by pepsin in acidic pH [11]. Briefly, 10 μg of protein in 20 mM Tris buffer, 150 mM NaCl, pH 8, were mixed with 400 μl of 0.5% trifluoroacetic acid and 100 μl of 8 M urea. Pepsin dissolved in water was added in an pepsin:protein ratio of 1:50. The digestion was performed for 2 h at room temperature. The resulting peptides were desalted on a C18 SPE column (PepClean, Thermo). During LC-MS/MS, the peptides were separated with a nano LC system (Ultimate 3000 RSLC nano, Dionex) on an AcclaimPepMap C18 column (75 um Internal Diameter, 250 mm length) using a 70 min elution gradient of acetonitrile in 0.1% formic acid. MS/MS data were acquired on a tandem mass spectrometer (TripleTOF 5600, Sciex) in a data-dependent manner selecting up to 15 precursor ions for fragmentation in each cycle. The resulting raw data were searched against a database consisting of mycobacterial proteins as well as proteins from *E*. *coli* and common contaminants in Protein Pilot 4.5 (Sciex). Ions suspected of containing disulfides were subjected to manual curation to confirm the position of the disulfide bridge within the peptide molecule.

### Molecular dynamics

#### System preparation

The prepared and optimized Pck-GDP-Mn^2+^ complex from our previous work [10] was used. For this analysis, we removed the cofactor to match the mass spectrometry conditions. Likewise, we discarded the Mn^2+^ ion, which has a negligible effect on the structure [10], and the water molecules. A truncated octahedron of explicit TIP3P water molecules, which extended 11 Å from the protein, was added in the Leap module of AMBER14 [12]. Nineteen Na^+^ counterions were put in places with the highest electrostatic potential to maintain the electroneutrality of the system. The AMBER ff14SB force field [13] was used for the protein, and Joung and Cheatham parameters [14] for the Na^+^ ions. The added waters and counterions were relaxed using the published protocol [15].

#### Simulations

After the system was gradually heated to 300 K over 50 ps and equilibrated for 200 ps, a production run of 17 ns ensued, using previously published settings [15]. Thereafter, C391-C397 disulfide bond was formed using protocol we developed previously [16]. Briefly, 2 ns MD was run using harmonic restraints of 1, 5, 10, 20, 30, 40, and 50 kcal/mol/Å on the distance between the side chain sulfurs (SG…SG) with a target of 3.6 Å (noncovalent SG…SG distance). For the selected restraint strength of 20 kcal/mol/Å, the two HG hydrogens were deleted and the disulfide bond formed. The waters and counterions were removed, and the 5 Å surroundings of the two cysteines were optimized. Waters and counterions were added again, and the system was run through relaxation, warming, equilibration, and production MD of 29 ns with the same settings as detailed above.

### Circular dichroism and Raman spectroscopy

The circular dichroism (CD) spectra of wt MTb Pck and the mutants were measured separately in the farUV (190–260 nm) and near UV (250–320 nm) spectral regions in standard experimental setups: far-UV, 0.1-cm quartz cells, protein concentration of 41 μM, 2 scans, 0.1-nm steps, 10 nm/min speed, 16 sec time constant, 1-nm spectral bandwidth; near UV, 0.2-cm quartz cells, protein concentration of 410 μM, 2 scans, 0.1-nm steps, 10 nm/min speed, 16 sec time constant, 1-nm spectral bandwidth. All samples were in 20 mM Tris–HCl, 150 mM NaCl, pH 7.4. Following baseline correction, the final spectra were expressed as molar ellipticity, (deg cm^2^ dmol^-1^) per residue.

Raman spectra were obtained using the drop coating deposition Raman spectroscopy technique [17]. For this purpose, samples with the same concentrations as for CD in 20 mM Tris-HCl buffer, pH 7.5, were dialyzed (Millipore filters 0.025 μm/white, VSWP/13 mm) against distilled water (30 min). The original and dialyzed samples were deposited (2 μL) on CaF_2_ slides and dried in air at room temperature (20°C for approximately 20 min).

Subsequently, Raman spectra were measured using an HR800 Raman microspectrometer (Horiba Jobin Yvon) with a 514.53 nm Ar^+^-ion excitation laser (10 mW) for the 1800–3300 cm^–1^ wavenumber interval. A 50× microscope objective (N.A. 0.75, Olympus) was used to focus the excitation laser on the sample to a diameter of approximately 1.5 μm. The spectra were collected using 600 grooves/mm grating and a liquid nitrogen-cooled CCD detector (1024×256 pixels, Symphony) with an integration time of 10×120 sec for He-Ne excitation and 10×480 sec for Ar^+^-ion excitation. The spectrometer was calibrated using Si-vibrations (at 520.7 cm^–1^) and a neon-glow lamp. The spectral resolution was approximately 5 cm^–1^. The Raman spectrum of the CaF_2_ substrate was carefully subtracted and background corrected. The Raman spectra in the S-H stretching region were normalized to the bands area.

## Results

### The disulfide bridge is formed by C391 and C397 in MTb Pck

**Fig 1:**
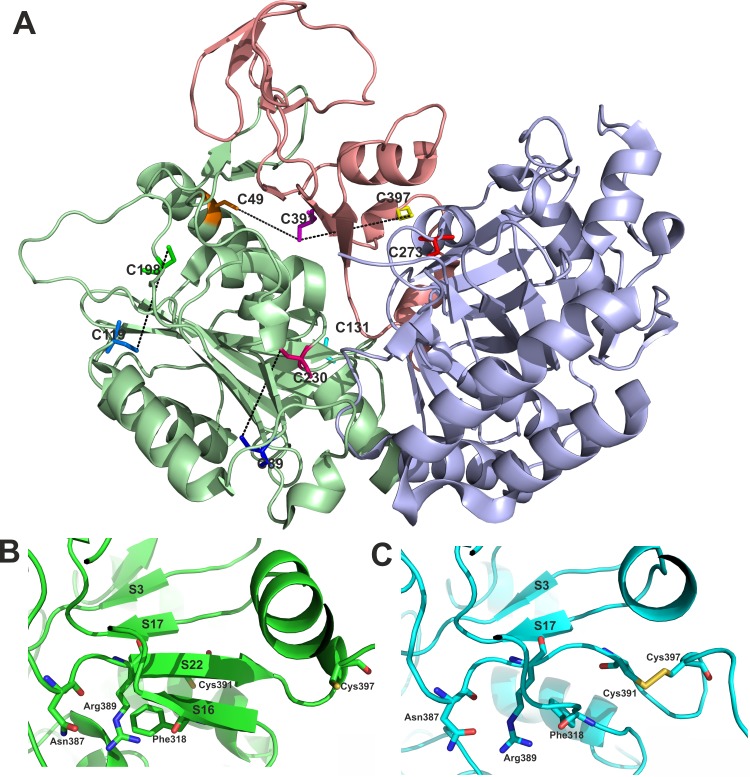
Cysteine residues in MTb Pck. (A) Cys residue distribution in Pck. Cys residues are shown as sticks, and the potential S–S bridges are marked by dotted lines. The criterion for selection was a Cys–Cys distance less than 15.0 Å in the X-ray structure of reduced form of MTb Pck (PDB code: 4R43) [10]. (B) Position and orientation of C391, C397 in the X-ray structure of reduced wt Pck[10]. C391 is localized in S22 β-strand. (C) The local structural changes in S22 and S16 β-strands in the C391-C397 disulfide containing Pck model prepared by molecular dynamics simulation. Color coding of sticks: blue–nitrogen, red–oxygen, yellow–sulfur.

**Fig 2:**
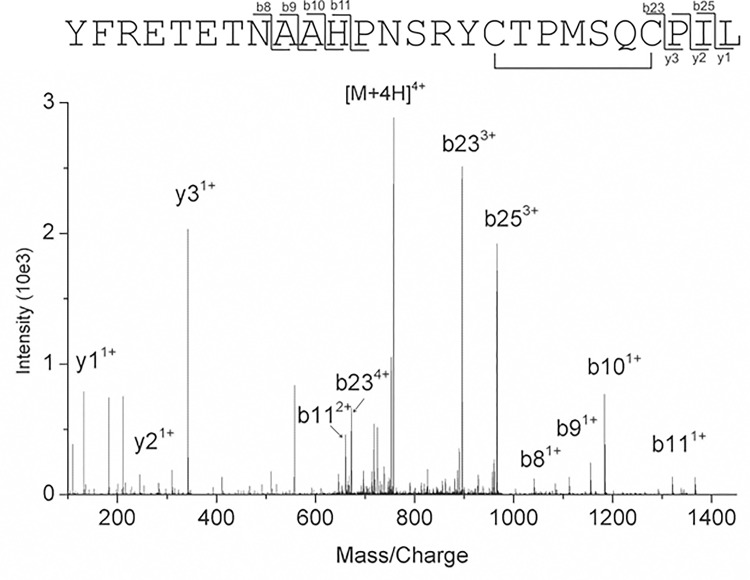
MS/MS spectrum of ion m/z 757.6. The experimental fragment ions correspond to the theoretical fragmentation of peptide YFRETETNAAHPNSRYCTPMSQCPIL with the cysteines C391 and C397 in the form of disulfide bridge. Most intense fragments visible in the spectrum are annotated by specific fragmentation series, number of the fragment in the series and the charge of the ion (in exponent). The same fragments are also visualized in the peptide sequence on top of the spectrum. For details of the fragmentation, see the S1 Table.

The distance measurement between all possible cysteine pairs in the X-ray crystal structure of reduced form of wt Pck in complex with GDP and Mn^2+^ (PDB 4RCG; 4R43) [10] indicates four possible S-S bridge variants (Fig 1A) with Cys–Cys distances not exceeding 15.0 Å: C119–C198 (13.6 Å), C391–C49 (10.5 Å), C230–C89 (12.3 Å), and C391–C397 (14.4 Å). To confirm presence of disulfides, we performed LC-MS/MS analysis of pepsin digest of Pck sample without addition of reducing agent. Prior to digestion, we alkylated cysteine thiol groups by iodoacetamide, which resulted in carbamidomethylation of cysteines and increasing the mass of each cysteine residue by 57.02 mass units (S57). All cysteines in the protein molecule, including C391 and C397, were alkylated by iodoacetamide. Only C391 and C397 also were found in the oxidized form of a disulfide bridge. We identified several peptides containing a disulfide bond between C391 and C397. Fig 2 shows corresponding MS/MS fragmentation spectrum of peptide YFRETETNAAHPNSRYCTPMSQCPIL. The list of identified peptide fragments highlighted as compare to all theoretical fragment ions are listed in S1 Table.

We further investigated the impact of C391-C397 bridge formation on local conformation changes in Pck structure. The crystal structure of reduced Pck form [10] shows that C391 is oriented to the opposite direction than C397, and is located in the S22 β-strand, which is part of five-stranded β-sheet (Fig 1B). All efforts to get crystals of suitable quality for X-ray structure determination of oxidized Pck containing S-S bridge were not successful and therefore we used restrained molecular dynamics (MD) to simulate the local structural rearrangements in Pck upon C391-C397 disulfide bridge formation. The S-S bridged Pck model was prepared using the force constant of 20 kcal/mol/A as described in more details in Material and Methods. According to the model, the S22 as well as the adjacent S16 β-strands loosed their secondary structure and orientations of Asn 387, Arg 389, and Phe 318 side chains were changed (Fig 1C) during the S-S bridge formation.

### Influence of C391 and C397 on Pck structure stability

**Fig 3:**
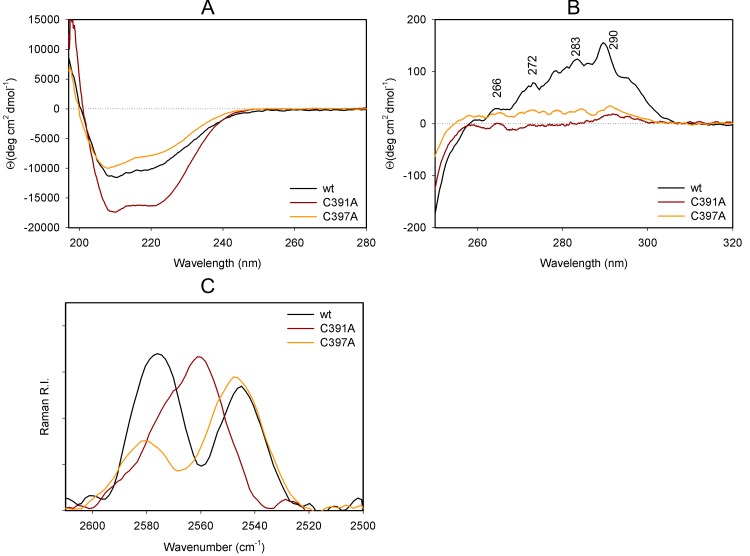
Analysis of secondary and tertiary structure changes in Pck Cys mutants. Far UV CD (A) and near UV CD (B) spectra of wt Pck and cysteine mutants (wt Pck–black, Cys391 mutants–dark red, and Cys397 mutants–orange. Raman spectra of wt Pck and Pck mutants in the cysteine S–H stretching vibrations region (2520 to 2600 cm^-1^) (C). wt Pck–black, Cys391 mutants–dark red, and Cys397 mutants–orange.

To determine contribution of C391 and C397 to Pck structure stability, we mutated these Cys residues to the non-polar Ala residue and used the circular dichroism (CD) spectroscopy in the far-UV (195 nm– 280 nm) and near-UV spectral regions for analysis of secondary and tertiary structure changes, respectively. The obtained far-UV spectra of C397A sample without addition of reducing agents revealed only small secondary structure changes compared to the wt Pck measured under identical conditions. We observed more profound secondary structure changes caused by the increase of α-helical conformation for C391 mutant (Fig 3A, Table 2). These data suggest that formation of the disulfide in MTb Pck can cause only local conformational changes but the overall protein structure is not changed.

**Table 2:** Distribution of secondary structure motifs in wt and mutated Pck based on circular dichroism measurements.

	α-helix	β-sheet	β-turn	random coil
wt	31%	19%	22%	28%
C391A	47%	9%	22%	24%
C397A	26%	23%	22%	30%

The near-UV CD spectra revealed the importance of C391 and C397 for stabilization of tertiary structure of Pck, as mutation of these residues caused significant spectral changes (Fig 3B). The CD bands typical for aromatic side chains of Trp (290 nm), Tyr (283 nm, 278 nm and 272 nm) and Phe (265, 258 nm) [18] were present in spectra of all the studied proteins however, the overall spectral intensity was markedly higher for wt Pck.

To investigate the local SH structure and dynamics in wt Pck and the mutants, we used Raman spectroscopy and analyzed the sulfhydryl stretching vibration spectral region (2500–2600 cm^-1^) [19]. For wt Pck and C397A mutant, the Raman S–H stretching bands occurred at 2575 cm^-1^ and 2545 cm^-1^, on the other hand for C391A mutant, these bands occurred at 2560 cm^-1^ with a shoulder at 2570 cm^-1^ (Fig 3C). Such spectral differences indicate that the S–H group of C391 behaves as strong hydrogen donor [19] and can widely interact with amino acids in the environment.

### Mutation of C391 and C397 modifies the activity of MTb Pck

**Fig 4:**
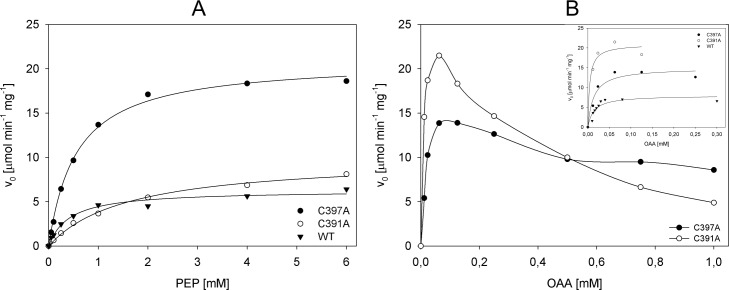
Substrate kinetics for C391A and C397A Pck mutants. (A) OAA synthesis via anaplerotic reaction and (B) PEP synthesis via gluconeogenic reaction.

We measured and compared activities of the C391A, and C397A mutants with wt Pck in both, anaplerotic and gluconeogenic reactions. The catalytic efficiency of C391A in the anaplerotic reaction decreased more than twice, namely due to the significantly increased K_m_ value (Fig 4A, Table 3), which corresponds well with the impaired secondary and tertiary structure of this mutant detected by CD and Raman spectroscopy. The K_m_ value of C397A in the anaplerotic reaction slightly increased and local rearrangement caused by mutation of this Cys residue was probably more convenient for anaplerotic reaction, since the value of maximal velocity V_max_ increased almost four times, which resulted in increased catalytic efficiency of this mutant. In gluconeogenic reaction we observed inhibition of reaction by increasing concentration of substrate for both mutants. Concentration of OAA exceeding 0.125 μM (C391A) and 0.25 μM (C397A) inhibited biosynthesis of PEP (Fig 4B) in contrast to wt Pck of which the activity was constant up to 1 mM OAA [9]. These data indicate that C391 and C397 residues are important for proper catalytic function of Pck in both reactions.

**Table 3:** Kinetic values of wt and Pck cysteine mutants.

**Gluconeogenic reaction**			
**OAA**	**V**_**max**_	**K**_**m**_	**V**_**max**_**/K**_**m**_
	*μmol min*^*-1*^ *mg*^*-1*^	*μM*	
wt (0–0.3 mM)	8 ± 0.8	13.8 ± 4.6	0.6
C391A (0–0.125 mM)	21.0 ± 1.6	4.2 ± 2.3	5
C397A (0–0.25 mM)	15.0 ± 1.3	13.6 ± 5.2	1.1
**Anaplerotic reaction**			
**PEP**	**V**_**max**_	**K**_**m**_	**V**_**max**_**/K**_**m**_
	*μmol min*^*-1*^ *mg*^*-1*^	*μM*	
wt (0.05–6 mM)	6.3 ± 0.3	414.1 ±77.29	0.015
C391A (0.05–6 mM)	10.0 ± 0.3	1605.6 ± 148.1	0.006
C397A (0.05–6 mM)	20.9 ± 0.4	559.2 ± 37.9	0.037

## Discussion

MTb Pck is a key enzyme in carbon metabolism, interconnecting glycolysis, gluconeogenesis, and the tricarboxylic acid cycle through the PEP-pyruvate-OAA node. Carbon flux through this node can be regulated by cellular conditions, substrate accessibility, enzyme activities, and enzyme inhibition by metabolites. In most microorganisms and mammals, Pck preferentially catalyzes the gluconeogenic reaction, because the concentration of OAA and the reaction thermodynamics favor production of PEP for building of sugar backbones during biosynthesis of nucleotides and several amino acids [20]. In growing MTb, Pck preferentially catalyzes gluconeogenic reaction however in non-replicating MTb, Pck function is important for carbon fixation and anaplerotic production of OAA [8,9]. Previously, we showed that increased concentration of reducing agents, and upregulation of proteins maintaining a reduced intracellular state stimulate MTb Pck anaplerotic activity [9]. It remains, however, unclear how reducing conditions contribute to MTb Pck specificity. One reason could be a change in the redox state of the hyperreactive cysteine; another possibility could be a reduction of S–S bridges and resultant modulation of Pck structure and activity. The reactivity of cysteine residues has been studied in Pck from different species. Results have shown that low concentrations of exogenous thiols stabilize or optimize enzyme activity, whereas the presence of disulfides supports a loss of activity [21–24].

MTb Pck contains reactive Cys residue, involved in coordinating metal ions in the active site of GTP-dependent Pck family [25], in position 273. However, mutation of C273 decreases the gluconeogenic reaction and increases catalytic efficiency of MTb Pck [9], which suggests contribution of additional factors regulating MTb catalysis in different redox conditions.

Here, we investigated presence of disulfide bridge in MTb Pck and influence of Cys 391 and 397 on MTb Pck function. We indeed detected formation of disulfide between C391 and C397 in non-reducing conditions using mass spectrometry. Molecular dynamics simulations indicated that formation of C391-C397 disulfide resulted in local structural changes. MD simulation indicated that S-S bridge formation causes loosening of S16 and S22 β-strands, which in consequences orientation of Arg389, Asn387, and Phe318 side chains.

Mutation of C391 and C397 to Ala resulted also to impaired Pck activity and specificity. We obtained different results for C391A and C397A anaplerotic activities. Mutant C391A, having more impaired tertiary structure, was also significantly less efficient during anaplerotic reaction. Moreover, we observed inhibition of gluconeogenic reaction by increasing concentration of substrate for both mutants, suggesting changes of OAA binding in mutated MTb Pck. The X-ray structure of rat cytosolic Pck-OAA-Mn^2+^ complex (PDB code 2QF2) indicates that in catalytically competent Pck form OAA interacts with two argine residues (Arg87 and Arg405). Since the 3D structure of MTb Pck in the complex with OAA has not been determined yet, we used the structure of MTb Pck-oxalate (OXL, PDB code: 4WIU) and compared it with the rat Pck-OAA-Mn^2+^ complex structure (PDB code 2QF2) and found similar stereochemical orientation of OAA and OXL in both enzymes, which allowed us to use MTb Pck-OXL complex for structural information explaining consequences of Cys 391 mutation to Ala. This structure shows that OAA interacts with Arg 81 and Arg 389 and forms sandwich-like arrangement similar to that found in mammalian cytosolic Pck [26,27] (See S1 Fig). Mutation of Cys 391 can influence orientation of side chains of Arg 389 and its interaction with OAA. The low stability of both mutants has not allowed us to crystalize the C391A and C397A mutants for further detailed characterization.

Interestingly, we found that C391 and C397 in the reduced form play important role also for Pck tertiary stability. Mutation of C391 and C397 to Ala leads to destabilization of tertiary structure as documented by collected near-UV CD spectra and Raman spectroscopy analysis in the sulfhydryl stretching vibration spectral region (2500–2600 cm^-1^), which showed a difference in the reactivity of individual SH thiol group, particularly of C391.

Taken together, our study indicates importance of cysteine residues in positions 391 and 397 for Pck tertiary structure stability and enzyme specificity. Thus, a joint effect of the redox state of the hyper-reactive cysteine in position 273 [9] and the reactivity of SH thiol groups of cysteines 391 and 397, either forming a disulfide bridge or interacting with neighboring amino acids, influence the preference of Pck for anaplerotic reaction in MTb surviving in macrophages under increased reducing and hypoxia-triggered conditions.

## Supporting Information

S1 Fig The OAA position in sandwich-like arrangement with Arg 81 and Arg 389 in the active site of MTb Pck (PDB code:4WIU; yellow colour) and rat Pck (PDB code: 2FQ2; green colour). (DOCX)

S1 Table List of theoretical fragments of peptide YFRETETNAAHPNSRYCTPMSQCPIL in form of a disulfide bridge. The parent ion is 4+ charge ion of m/z = 757.5941. The table shows three fragment series (a, b, y) and three potential charge states (1+, 2+, 3+). Those ions that were identified in the spectrum with error less than 40 ppm are in bold and italic.
(DOCX)

## References

[1] Watanabe S, Zimmermann M, Goodwin MB, Sauer U, Barry CE, Boshoff HI. Fumarate reductase activity maintains an energized membrane in anaerobic Mycobacterium tuberculosis. PLoS Pathog. 2011;7: e1002287 10.1371/journal.ppat.1002287 21998585 PMC3188519

[2] Srinivasan V, Morowitz H. Ancient genes in contemporary persistent microbial pathogens. Biol Bull. 2006;2: 1–9. Available: http://www.biolbull.org/content/210/1/1.short 10.2307/4134531 16501059

[3] Rohde KH, Veiga DFT, Caldwell S, Balázsi G, Russell DG. Linking the transcriptional profiles and the physiological states of Mycobacterium tuberculosis during an extended intracellular infection. PLoS Pathog. 2012;8: e1002769 10.1371/journal.ppat.1002769 22737072 PMC3380936

[4] Kendall SL, Withers M, Soffair CN, Moreland NJ, Gurcha S, Sidders B, et al A highly conserved transcriptional repressor controls a large regulon involved in lipid degradation in Mycobacterium smegmatis and Mycobacterium tuberculosis. Mol Microbiol. 2007;65: 684–699. 10.1111/j.1365-2958.2007.05827.x 17635188 PMC1995591

[5] Shi L, Sohaskey CD, Kana BD, Dawes S, North RJ, Mizrahi V, et al Changes in energy metabolism of Mycobacterium tuberculosis in mouse lung and under in vitro conditions affecting aerobic respiration. Proc Natl Acad Sci U S A. 2005;102: 15629–15634. 10.1073/pnas.0507850102 16227431 PMC1255738

[6] Beste DJ V, Nöh K, Niedenführ S, Mendum TA, Hawkins ND, Ward JL, et al 13C-flux spectral analysis of host-pathogen metabolism reveals a mixed diet for intracellular Mycobacterium tuberculosis. Chem Biol. 2013;20: 1012–21. 10.1016/j.chembiol.2013.06.012 23911587 PMC3752972

[7] Shi L, Sohaskey CD, Pfeiffer C, Datta P, Parks M, McFadden J, et al Carbon flux rerouting during Mycobacterium tuberculosis growth arrest. Mol Microbiol. 2010;78: 1199–215. 10.1111/j.1365-2958.2010.07399.x 21091505 PMC3072047

[8] Beste DJ V, Bonde B, Hawkins N, Ward JL, Beale MH, Noack S, et al ^13^C metabolic flux analysis identifies an unusual route for pyruvate dissimilation in mycobacteria which requires isocitrate lyase and carbon dioxide fixation. Rubin EJ, editor. PLoS Pathog. 2011;7: e1002091 10.1371/journal.ppat.1002091 21814509 PMC3141028

[9] Machová I, Snášel J, Zimmermann M, Laubitz D, Plocinski P, Oehlmann W, et al Mycobacterium tuberculosis phosphoenolpyruvate carboxykinase is regulated by redox mechanisms and interaction with thioredoxin. J Biol Chem. 2014;289: 13066–78. 10.1074/jbc.M113.536748 24659783 PMC4036320

[10] Machová I, Snášel J, Dostál J, Brynda J, Fanfrlík J, Singh M, et al Structural and functional studies of phosphoenolpyruvate carboxykinase from Mycobacterium tuberculosis. Cardona P-J, editor. PLoS One. 2015;10: e0120682 10.1371/journal.pone.0120682 25798914 PMC4370629

[11] Liu F, van Breukelen B, Heck AJR. Facilitating protein disulfide mapping by a combination of pepsin digestion, electron transfer higher energy dissociation (EThcD), and a dedicated search algorithm SlinkS. Mol Cell Proteomics. 2014;13: 2776–86. 10.1074/mcp.O114.039057 24980484 PMC4189002

[12] Case DA, Darden T, Iii TEC, Simmerling C, Brook S, Roitberg A, et al Amber 14. Univ California, San Fr 2014

[13] Hornak V, Abel R, Okur A, Strockbine B, Roitberg A, Simmerling C. Comparison of multiple Amber force fields and development of improved protein backbone parameters. Proteins. 2006;65: 712–725. 10.1002/prot.21123 16981200 PMC4805110

[14] Joung IS, Cheatham TE. Determination of alkali and halide monovalent ion parameters for use in explicitly solvated biomolecular simulations. J Phys Chem B. 2008;112: 9020–41. 10.1021/jp8001614 18593145 PMC2652252

[15] Žáková L, Kletvíková E, Veverka V, Lepsík M, Watson CJ, Turkenburg JP, et al Structural integrity of the B24 site in human insulin is important for hormone functionality. J Biol Chem. 2013;288: 10230–40. 10.1074/jbc.M112.448050 23447530 PMC3624407

[16] Pytelková J, Lepšík M, Šanda M, Talacko P, Marešová L, Mareš M. Enzymatic activity and immunoreactivity of Aca s 4, an alpha-amylase allergen from the storage mite Acarus siro. BMC Biochem. 2012;13: 3 10.1186/1471-2091-13-3 22292590 PMC3306266

[17] Kopecký V, Baumruk V. Structure of the ring in drop coating deposited proteins and its implication for Raman spectroscopy of biomolecules. Vib Spectrosc. 2006;42: 184–187.

[18] Kelly SM, Jess TJ, Price NC. How to study proteins by circular dichroism. Biochim Biophys Acta. 2005;1751: 119–39. 10.1016/j.bbapap.2005.06.005 16027053

[19] Thomas GJ. New structural insights from Raman spectroscopy of proteins and their assemblies. Biopolymers. 2002;67: 214–225. 10.1002/bip.10105 12012434

[20] Sauer U, Eikmanns BJ. The PEP-pyruvate-oxaloacetate node as the switch point for carbon flux distribution in bacteria. FEMS Microbiol Rev. 2005;29: 765–94. 10.1016/j.femsre.2004.11.002 16102602

[21] Carlson GM, Colombo G, Lardy HA. A vicinal dithiol containing an essential cysteine in phosphoenolpyruvate carboxykinase (guanosine triphosphate) from cytosol of rat liver. Biochemistry. 1978;17: 5329–5338. 728403 10.1021/bi00618a002

[22] Lewis CT, Seyer JM, Cassell RG, Carlson GM. Identification of vicinal thiols of phosphoenolpyruvate carboxykinase (GTP). J Biol Chem. 1993;268: 1628–36. Available: http://www.ncbi.nlm.nih.gov/pubmed/8420937 8420937

[23] Makinen AL, Nowak T. A reactive cysteine in avian liver phosphoenolpyruvate carboxykinase. J Biol Chem. 1989;264: 12148–57. Available: http://www.ncbi.nlm.nih.gov/pubmed/2545699 2545699

[24] Krautwurst H, Encinas MV, Marcus F, Latshaw SP, Kemp RG, Frey P a, et al Saccharomyces cerevisiae phosphoenolpyruvate carboxykinase: revised amino acid sequence, site-directed mutagenesis, and microenvironment characteristics of cysteines 365 and 458. Biochemistry. 1995;34: 6382–8. 7756267 10.1021/bi00019a017

[25] Holyoak T, Sullivan SSM, Nowak T. Structural insights into the mechanism of PEPCK catalysis. Biochemistry. 2006;45: 8254–63. 10.1021/bi060269g 16819824

[26] Sullivan SM, Holyoak T. Structures of rat cytosolic PEPCK: insight into the mechanism of phosphorylation and decarboxylation of oxaloacetic acid. Biochemistry. 2007;46: 10078–88. 10.1021/bi701038x 17685635

[27] Carlson GM, Holyoak T. Structural insights into the mechanism of phosphoenolpyruvate carboxykinase catalysis. J Biol Chem. 2009;284: 27037–41. 10.1074/jbc.R109.040568 19638345 PMC2785633

