# Supplementary material for: The Role of Cysteine Residues in Catalysis of Phosphoenolpyruvate Carboxykinase from Mycobacterium tuberculosis
Source: PLoS One. 2017 Jan 30;12(1):e0170373. doi: 10.1371/journal.pone.0170373 (PMC5279734; doi:10.1371/journal.pone.0170373)
Supplement: S1 Fig — (DOCX) [file pone.0170373.s001.docx]

**S1 Fig.** The OAA position in sandwich-like arrangement with Arg 81 and Arg 389 in the active site of MTb Pck (PDB code:4WIU; yellow colour) and rat Pck (PDB code: 2FQ2; green colour).

**
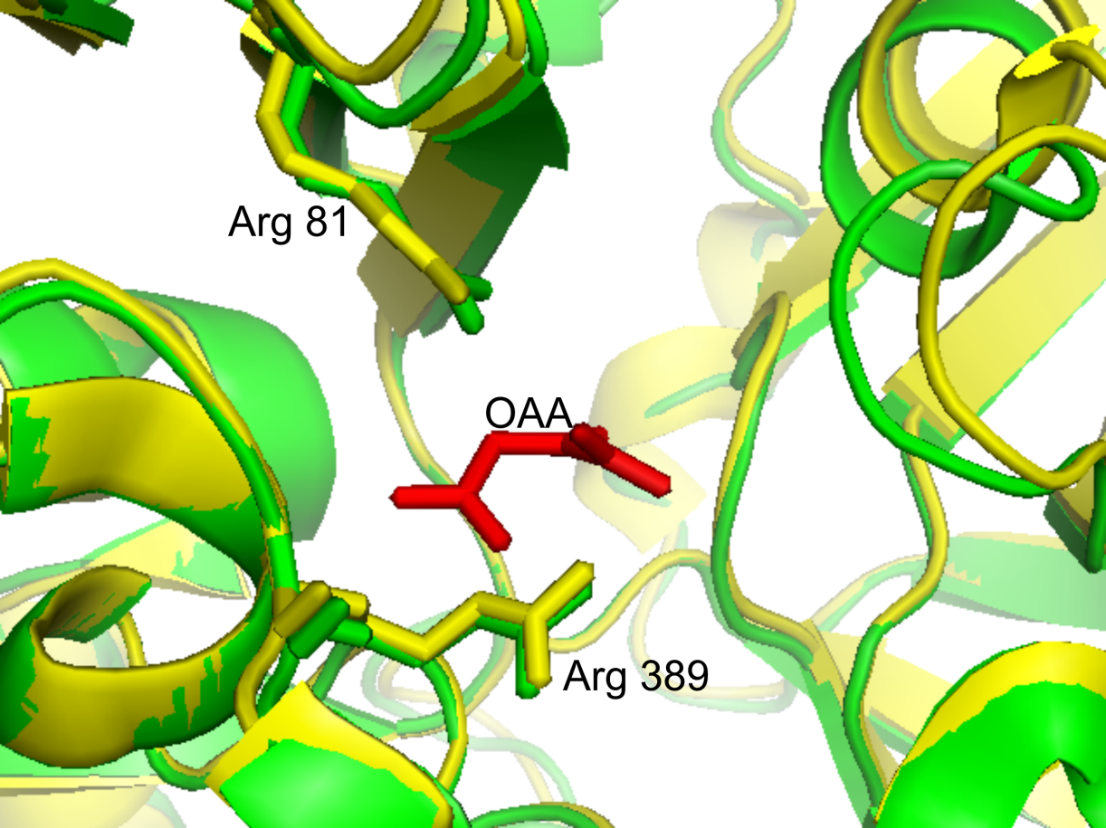
**
