# Supplementary material for: The Role of Cysteine Residues in Catalysis of Phosphoenolpyruvate Carboxykinase from Mycobacterium tuberculosis
Source: PLoS One. 2017 Jan 30;12(1):e0170373. doi: 10.1371/journal.pone.0170373 (PMC5279734; doi:10.1371/journal.pone.0170373)
Supplement: S1 Table — The parent ion is 4+ charge ion of m/z = 757.5941. The table shows three fragment series (a, b, y) and three potential charge states (1+, 2+, 3+). Those ions that were identified in the spectrum with error less than 40 ppm are in bold and italic. (DOCX) [file pone.0170373.s002.docx]

**S1 Table**: List of theoretical fragments of peptide YFRETETNAAHPNSRYCTPMSQCPIL in form of a disulfide

bridge. The parent ion is 4+ charge ion of m/z=757.5941. The table shows three fragment series (a, b, y) and three

potential charge states (1+, 2+, 3+). Those ions that were identified in the spectrum with error less than 40 ppm are

in bold and italics.

| Amino Acid | N - term | **a** |  |  | **b** |  |  | **y** |  |  | C-term |
| --- | --- | --- | --- | --- | --- | --- | --- | --- | --- | --- | --- |
|  | # | 1+ | 2+ | 3+ | 1+ | 2+ | 3+ | 1+ | 2+ | 3+ | # |
| Y | 1 | ***136,0757*** | 68,5415 | 46,0301 | 164,0706 | 82,5389 | 55,3617 | 3027,3547 | 1514,1810 | 1009,7897 | 26 |
| F | 2 | ***283,1441*** | 142,0757 | 95,0529 | ***311,1390*** | 156,0731 | 104,3845 | 2864,2913 | 1432,6493 | 955,4353 | 25 |
| R | 3 | 439,2452 | 220,1262 | 147,0866 | ***467,2401*** | 234,1237 | 156,4182 | 2717,2229 | 1359,1151 | 906,4125 | 24 |
| E | 4 | ***568,2878*** | 284,6475 | 190,1008 | ***596,2827*** | 298,6450 | 199,4324 | 2561,1218 | 1281,0645 | 854,3788 | 23 |
| T | 5 | ***669,3355*** | 335,1714 | 223,7833 | 697,3304 | 349,1688 | 233,1150 | 2432,0792 | 1216,5432 | 811,3646 | 22 |
| E | 6 | ***798,3781*** | ***399,6927*** | 266,7975 | 826,3730 | ***413,6901*** | 276,1292 | 2331,0315 | 1166,0194 | 777,6820 | 21 |
| T | 7 | 899,4258 | 450,2165 | 300,4801 | 927,4207 | ***464,2140*** | 309,8117 | 2201,9889 | 1101,4981 | 734,6678 | 20 |
| N | 8 | ***1013,4687*** | 507,2380 | 338,4944 | ***1041,4636*** | ***521,2354*** | 347,8261 | 2100,9413 | 1050,9743 | 700,9853 | 19 |
| A | 9 | ***1084,5058*** | ***542,7565*** | 362,1735 | ***1112,5007*** | ***556,7540*** | 371,5051 | 1986,8983 | 993,9528 | 662,9710 | 18 |
| A | 10 | ***1155,5429*** | 578,2751 | 385,8525 | ***1183,5378*** | 592,2726 | 395,1841 | 1915,8612 | ***958,4343*** | 639,2919 | 17 |
| H | 11 | ***1292,6018*** | ***646,8046*** | 431,5388 | ***1320,5967*** | ***660,8020*** | 440,8704 | 1844,8241 | 922,9157 | 615,6129 | 16 |
| P | 12 | 1389,6546 | 695,3309 | 463,8897 | 1417,6495 | 709,3284 | 473,2214 | 1707,7652 | ***854,3862*** | 569,9266 | 15 |
| N | 13 | 1503,6975 | ***752,3524*** | 501,9040 | 1531,6924 | ***766,3499*** | 511,2357 | 1610,7124 | 805,8599 | 537,5757 | 14 |
| S | 14 | 1590,7295 | 795,8684 | 530,9147 | 1618,7245 | 809,8659 | 540,2463 | 1496,6695 | 748,8384 | 499,5614 | 13 |
| R | 15 | 1746,8307 | 873,9190 | 582,9484 | 1774,8256 | ***887,9164*** | ***592,2800*** | 1409,6375 | 705,3224 | 470,5507 | 12 |
| Y | 16 | 1909,8940 | 955,4506 | 637,3028 | 1937,8889 | ***969,4481*** | 646,6345 | 1253,5364 | 627,2718 | 418,5170 | 11 |
| C[-1.0078] | 17 | 2011,8954 | 1006,4513 | 671,3033 | 2039,8903 | 1020,4488 | 680,6349 | 1090,4730 | 545,7402 | 364,1625 | 10 |
| T | 18 | 2112,9431 | 1056,9752 | 704,9859 | 2140,9380 | 1070,9726 | 714,3175 | 988,4717 | 494,7395 | 330,1621 | 9 |
| P | 19 | 2209,9958 | 1105,5015 | 737,3368 | 2237,9907 | 1119,4990 | 746,6684 | 887,4240 | 444,2156 | 296,4795 | 8 |
| M | 20 | 2341,0363 | 1171,0218 | 781,0170 | 2369,0312 | 1185,0192 | 790,3486 | 790,3712 | 395,6892 | 264,1286 | 7 |
| S | 21 | 2428,0683 | 1214,5378 | 810,0276 | 2456,0632 | 1228,5353 | 819,3593 | 659,3307 | 330,1690 | 220,4484 | 6 |
| Q | 22 | 2556,1269 | 1278,5671 | 852,7138 | 2584,1218 | 1292,5645 | 862,0455 | 572,2987 | 286,6530 | 191,4377 | 5 |
| C[-1.0078] | 23 | 2658,1283 | 1329,5678 | ***886,7143*** | 2686,1232 | ***1343,5652*** | ***896,0459*** | 444,2401 | 222,6237 | 148,7516 | 4 |
| P | 24 | 2755,1811 | 1378,0942 | ***919,0652*** | 2783,1760 | 1392,0916 | ***928,3968*** | ***342,2387*** | 171,6230 | 114,7511 | 3 |
| I | 25 | 2868,2651 | 1434,6362 | ***956,7599*** | 2896,2600 | 1448,6337 | ***966,0915*** | ***245,1860*** | 123,0966 | 82,4002 | 2 |
| L | 26 | 2981,3492 | 1491,1782 | 994,4546 | 3009,3441 | 1505,1757 | 1003,7862 | ***132,1019*** | 66,5546 | 44,7055 | 1 |
